# Targeting GPCRs Against Cardiotoxicity Induced by Anticancer Treatments

**DOI:** 10.3389/fcvm.2019.00194

**Published:** 2020-01-24

**Authors:** Anais Audebrand, Laurent Désaubry, Canan G. Nebigil

**Affiliations:** Laboratory of CardioOncology and Therapeutic Innovation, CNRS, Illkirch, France

**Keywords:** GPCRs, cardiotoxicity, melatonin, ghrelin, galanin, apelin, prokineticin, cannabidiol

## Abstract

Novel anticancer medicines, including targeted therapies and immune checkpoint inhibitors, have greatly improved the management of cancers. However, both conventional and new anticancer treatments induce cardiac adverse effects, which remain a critical issue in clinic. Cardiotoxicity induced by anti-cancer treatments compromise vasospastic and thromboembolic ischemia, dysrhythmia, hypertension, myocarditis, and cardiac dysfunction that can result in heart failure. Importantly, none of the strategies to prevent cardiotoxicity from anticancer therapies is completely safe and satisfactory. Certain clinically used cardioprotective drugs can even contribute to cancer induction. Since G protein coupled receptors (GPCRs) are target of forty percent of clinically used drugs, here we discuss the newly identified cardioprotective agents that bind GPCRs of adrenalin, adenosine, melatonin, ghrelin, galanin, apelin, prokineticin and cannabidiol. We hope to provoke further drug development studies considering these GPCRs as potential targets to be translated to treatment of human heart failure induced by anticancer drugs.

## Introduction

New anticancer treatments have improved overall mortality (1). However, most of the anticancer drugs display a wide array of cardiovascular toxicities, leading to interruption of cancer therapies and maladaptive remodeling in hearts, affecting the short- and long-term quality of life (2–4). Oxidative stress and inflammation are inter-reliant processes involved in cardiovascular diseases and cancers (5, 6), along with apoptosis (7, 8) and necrosis (9). Tissue resident and circulating inflammatory cells (such as macrophages, mast cells, neutrophils, and monocytes) can also release both reactive oxygen species (ROS) and reactive nitrogen species (RNS) to induce an oxidative stress (6). Due to negligible detoxification capacity, the heart is particularly susceptible to ROS and RNS injury (10). Thus, high levels of ROS and RNS can debilitate cardiac cellular signaling pathways and can augment the gene expression of proinflammatory (11) and antioxidant defenses as the major cause for necrosis and apoptosis.

Classic chemotherapeutics particularly anthracyclines are the prototype of drugs causing cardiotoxicity (12). They can induce acute cardiotoxicity, including reversible hypotension, pericarditis and transient electrocardiographic abnormalities (changes in the ST-T waves, QT prolongation), and vasodilatation (13). However, after completion of cumulative dose regimens, anthracyclines promote irreversible cardiomyopathy (classified as type (1) cardiotoxicity), leading to heart failure (HF) (13, 14). Doxorubicin (DOX), the most frequently used anthracyclines can cause irreversible type 1 cardiotoxicity via accumulation of ROS and RNS (15, 16). They also target Topoisomerase IIβ (Top IIβ) in cardiomyocytes to induce DNA damage and apoptosis. Recently, the anthracycline mediated cardiotoxicity has been reviewed by Nebigil (17).

Targeted therapies also provoke some degree of cardiotoxicity. Targeting key tyrosine kinases (TKs) with TK antibodies and inhibitors has a remarkable achievement in cancer management. However, they also induce cardiotoxicity, because they block pathways that also regulate myocardial function (18). This cardiotoxicity is often reversible, and thus classified as type 2 cardiotoxicity (19, 20). It results in ultrastructural changes in cardiomyocytes, with reversible cardiac dysfunctions such as elevated blood pressure, thromboembolism, pericardial thickening, and arrhythmia (21). Type 1 and 2 forms of cardiotoxicity can overlap, when the classic and targeted therapeutics used together or subsequently. For example, in patient treated with anthracyclines earlier, trastuzumab, a monoclonal antibody anti-HER-2 can cause irreversible cardiac damage and left ventricular (LV) dysfunction (18, 22, 23). On the other hand, 27%, of patients who received both anthracycline and trastuzumab encountered cardiac dysfunction, while this rate was of 2-16% for patients treated with anthracyclines alone (24).

Recent studies have demonstrated that patients treated with immune checkpoint inhibitors (25) also develop myocarditis due to immune-related adverse events (6, 26). The therapeutic mechanisms of inhibitors mostly rely on blocking either the cytotoxic T-lymphocyte associated antigen-4 (CTLA-4) or programmed cell death protein-1 (PD-1) pathways, while activating the host's immune system against cancer (27). CTLA-4 and PD-1 act as immune response inhibitors (6, 28). They suppress the T-cell response in order to prevent autoimmunity and maintain T-cell tolerance. Cardiac immune-related adverse events appear more frequently in patients treated with CTLA-4 antagonists compared with PD-1 inhibitors (29) and the myocarditis risk increases with combination therapy, leading to discontinuation in approximately 50% of patients (30, 31) probably due to targeting PD-1 and CTLA-4 in cardiomyocytes as well.

## Clinically Used Cardioprotective Agents Against Cardiotoxicity

There are several cardioprotective therapeutics that have been used against anticancer-mediated cardiotoxicity. Their properties are summarized in Table 1.

**Table 1:** Prophylactic cardioprotective agents.

**Clinically used cardioprotective agents**	**Mechanism of cardioprotection**	**Name of molecules**	**Anti-tumor effect**	**Study limitations**
**Antioxidants**	⇓ROS and RNS (32–34)	Vitamin C (35) Resveratrol (36) Bicalein (37)	A risk of loss of oncological efficacy	No improvement in survival rate (32)
**Dexrazoxane**	Iron chelator and detoxifying agent, ⇓ Topoisomerase Iiβ (25, 38–41)	Topotect Zinecard Cardioxane	It increases risk of infection and myelosuppression second primary malignancies, leukopenia (78%) (40)	No improvement in survival rate (39)
**Statin**	⇑Vasodilatation, anticoagulation, ⇓platelet, antioxidant and anti-inflammatory functions; ⇓Topoisomerase II via Rac1 inhibition (42–45)	Lipitor Simvastatin Lovastatin Zocor Lescol Crestor Livalo	The meta-analyses suggested that statin can reduce cancer (expecially breast cancer)-mediated mortality (46)	40% patients use ACEIs and β-blockers together with statin, thus it is difficult to estimate the cardioprotective effectiveness of statin. Decreasing synthesis of mevalonic acid It can lead to muscle injury and diabetes (47)
**Beta-AR blokers** β1-AR acts through Gs and Ca^2+^/calmodulin-dependent protein kinase (CaMKII) β2-AR acts through the Gi and Akt pathway	⇓ROS generation ⇓Apoptosis in cardiomyocyte ⇓Mitochondrial complex-I (carvedilol)(48, 49) and vasodilatory effects (nebivolol) (50)	Carvedilol Nebivolol Metoprolol	The role of β-blockers on cancer-specific survival rate resulted in conflicting results (51, 52)	The benefit of the use of prophylactic beta-blockers for prevention of chemo-induced cardiotoxicity remains unclear (53). The non-selective β1 and β2 blockers could be more beneficial due to antioxidant effects (28)
**ACEIs and angiotensin receptor blokers** AT1R uses G_q/11_, G_i_, G_12_ and G_13_ coupled to PLCβ and Rho/ROCK. ⇑ROS generation, transactivation of growth factor receptors (IGF-1R).	⇓Vasoconstriction, ⇓Inflammation, ⇓Fibrosis, ⇓Hypertrophy ⇓Catecholamine and aldosterone release (54, 55)	Valsartan Candesartan Cilexetil	Antitumor effect is conflicting (56, 57)	Human trials are not conclusive yet. Combination of enalapril with metoprolol or candesartan has no clear beneficial effects (48)

### Antioxidants

Beneficial effects of antioxidants on LV remodeling and amelioration of contractility have been demonstrated in many experimental models of HF. For example, vitamin C effectively mitigates DOX-induced oxidative stress and apoptosis in rats (35). Resveratrol, a polyphenolic compound has also both prophylactic and therapeutic benefits in reversing DOX induced apoptosis and fibrosis in rat myocardium (36). Baicalein, a bioflavonoid can alleviate cardiotoxicity in mice (37). However, elimination of ROS and RNS by antioxidant drugs may be detrimental and even impair physiological cellular functions (58). There is also a risk of loss of oncological efficacy, because of the overlapping mechanisms with cardioprotective effects. Nevertheless, in clinic these approaches did not significantly improve survival rate and they may even increase mortality if they do not have other pharmacological properties (32, 59).

### Dexrazoxane

Dexrazoxane is an iron chelator and detoxifying agent that can prevent anthracycline-associated cardiotoxicity. It also acts on Topoisomerase IIβ to promote cardioprotective effects. Dexrazoxane is the only Food and Drug Administration (FDA) and the European Medicines Agency (EMA) approved cardioprotective drug to against chemotherapeutics-mediated HF (38, 60). However, its use in children and adolescent were forbidden by EMA in 2011, because it increases risk of infection, myelosuppression and second primary malignancies. These restrictions by EMA have been partially altered based on the new findings in 2018 (39). Only use of dexrazoxane was allowed in patients who have received a cumulative DOX at the dose of 300 mg/m (2) and are continuing with this medicine. Although dexrazoxane is a valuable option to prevent cardiotoxicity, it induces a severe leukopenia in 78% of cancer patients (40). Use of dexrazoxane is not recommended with non-anthracycline chemotherapy regimens.

### Statin

Statins are used to lower low-density lipoprotein (LDL) and cholesterol amount in the blood on patients suffering to arterosclerosis (61). The mechanism involved in this action is due to inhibition of HMG-CoA reductase, which is involved the biosynthesis of cholesterol. Statins also display significant vasodilatation, platelet inhibition, anti-inflammatory, and antioxidant effects due to their pleiotropic effects (62, 63). Statin (atorvastatin) could be effective in maintenance of LV ejection fraction (LVEF) in patients treated with anthracycline (42). Moreover, it could limit oxidative stress and vascular inflammation (64) and activate autophagy (43) to promote cardioprotective effects against dasatinib. Statins also inhibits Top IIβ mediated DNA damage via Rac1 inhibition. Recent meta-analyses suggest that statins are at least equally potent as dexrazoxane in the prevention of anthracycline-induced cardiotoxicity (65). Calvillo-Argüelles and colleagues have found that in HER2^+^ breast cancer patients treated with trastuzumab with or without anthracycline, the concomitant statin use was associated with a lower risk of cardiotoxicity (44). Although, several studies on the influence of statin therapy on development of cancer risk resulted in conflicting results, the recent meta-analyses suggested that statin can reduce cancer-mediated mortality (46). However, there are some studies show that statin induces myopathies that may be due to decreased synthesis of mevalonic acid, leading to decreased energy generation and muscle injury. Another side effect associated with statin usage is new-onset diabetes (47). Many of the beneficial effects of a statin is due to inhibition of heterotrimeric G proteins, including Ras and Rho or Rac1 signaling (45). Thus, the specific Rho and Rac inhibitors may be more preferable targets for future chemo-preventive strategies.

### GPCRs

As seven transmembrane (7TM) domain proteins, G protein-coupled receptors (GPCRs) represent the largest family of cell surface proteins (66). GPCRs regulate many physiological processes in every tissue, making the GPCR superfamily a major target for therapeutic intervention (67). The binding of agonists to GPCRs not only initiates the “classical,” signaling cascades through heterotrimeric G proteins (composed of the three subunits, Gα, Gβ, and Gγ). It can also activate G-protein-independent pathways involving β-arrestin (68, 69). Indeed, β-arrestins are identified as scaffolding proteins for MAP kinases and serine/threonine kinases cascades (70). The discovery that some GPCRs prefer to activate G-protein- or arrestin-mediated pathways has given rise to efforts to produce signal biased drugs (71). The drug discovery efforts aim to produce “biased” and/or allosteric ligands with less adverse effects without compromising their efficacy (72). In cardiovascular system, GPCRs can lead to hypertrophy, apoptosis, contraction, and cardiomyocytes survival. Some of the GPCR targeted therapeutics are used in clinic for treatment of heart failure and cardiotoxicity (Table 1).

### Preventive and Prophylactic Strategies Targeting GPCRs Against Anticancer-Induced Cardiotoxicity

#### β-Blockers

β-adrenergic receptors (β-ARs) play a crucial role in cardiovascular regulation. It exists 3 types of β-ARs: β_1_, β_2_ and β_3_. Cardiac adrenergic receptor corresponding to β_1_-ARs whereas β_2_-ARs are localized on blood vessels. β_1_-ARs, are coupled to the Gα_s_ and activate adenylyl cyclase to exert a positive inotropic, chronotropic and dromotropic effects in the heart. Indeed, β_1_-ARs increase heart rate, cardiac contractility and myocardial oxygen demand, thus promoting myocardial ischemia in patients with coronary heart disease. More importantly, persistent β_1_-ARs induce myocyte apoptosis and hypertrophy by activating CaMKII. On the opposite, persistent β_2_-ARs activation protects myocardium through a Gαi-mediated pathway, and activating PI3K, and Akt kinase probably via small G proteins (73). Administration of β2-AR agonist and β1-AR antagonist seems to be better than β2-AR antagonist in HF prevention. Interestingly, β3-AR is activated by catecholamines at higher concentration than those required to activate β1-AR and β2-AR (73). Thus, β3-AR plays an important protective role in the cardiovascular system during sympathetic over-stimulation.

It exists three mains β-AR blockers. The first generation of β*-*blockers, such as propranolol, inhibits both β_1_ and β_2_-ARs. The second generation of β-blockers (metoprolol) are cardioselective (β_1_-ARs).

The third generation of β-blockers (carvedilol and nebivolol) are vasodilators that not only inhibit β1 and α_1_-adrenoreceptors, but they also activate β_3_-adrenergic receptors (74). Carvedilol also reduces ROS generation and apoptosis in cardiomyocyte (49). Nebivolol has a vasodilatory effect mediated by nitric oxide release and avoid vasoconstriction to decrease blood pressure in hypertensive patients (50). Two clinical studies showed that carvedilol prevent cardiotoxicity in female patients diagnosed with breast cancer (75, 76). This cardioprotective effects has been attributed to its antioxidant and anti-apoptotic properties rather than its β*-*AR blocking activity, because carvedilol inhibits mitochondrial complex-I that promotes cardiotoxicity (77). This cardioprotective effect of carvedilol is superior than metoprolol and atenolol for preventing cardiomyocytes against DOX-induced apoptosis (78). In contrast, Avila and his colleague showed that carvedilol has no impact on the LVEF reduction induced by anthracycline in breast cancer patients (53). The recent meta-analyses on cancer patients have demonstrated that the use of β-blockers is not associated with cancer prognosis (51). Indeed, several studies on the influence of β-blockers on cancer-specific survival rate resulted in conflicting results (51, 52). The beneficial effects of non-selective β1 and β2 blockers could be due to their antioxidant effects (28).

#### Angiotensin Converting Enzyme Inhibitors (ACEI) and Angiotensin (AngII) Receptor Blockers (ARB)

Renin-angiotensin-aldosterone (RAAS) system regulates the cardiac and renal functions. Ang-II interacts with two GPCRs: AT-1R and AT-2R that are associated with opposite functions (79). However, most of the effects of renin-angiotensin system (RAS) are mediated by AT-1R, which promotes vasoconstriction, inflammation, fibrosis, hypertrophy, and releasing of catecholamine and aldosterone. AT-2 is implicated to vasodilatations, inhibition on cell growth, apoptosis, and bradykinin releasing. Increasing of Ang-II also stimulates sympathetic system and the production of aldosterone, leading to LV hypertrophy (80). Reduction of excessive Ang-II and aldosterone decrease cardiovascular morbidity and mortality. Indeed, AT-1R blockers ACE inhibitors are of paramount importance in treatment of cardiovascular diseases, including hypertension (54).

Several clinical trials indicate that Angiotensin-II receptor blockers (ARB) alleviate anthracycline cardiotoxicity (55), however, prospective trials are still needed for further validation. The expression of AngII and AT-1R have been found in many cell types of the tumor microenvironment (56). Thus, the RAS may alter remodeling of the tumor microenvironment and the immuno-suppressive milieu, thereby affecting tumor growth. In contrast, meta-analysis derived from the results of a group of trials demonstrated that ARB may promote the occurrences of new tumors (especially lung cancer) (57). These findings warrant further investigation.

The cardioprotective effects of combined ACEIs/ARBs and β-blockers have been evaluated during anthracycline, trastuzumab, or sequential chemotherapy. The combination of carvedilol and enalapril has been shown to preserve the LV function in adult patients treated with anthracyclines (81). However, other trials with combination of enalapril with metoprolol (82) or candesartan with metoprolol (83), ended up with disappointing results. Indeed, Guglin and his colleague recently demonstrated that both lisinopril and carvedilol do not prevent the cardiotoxicity of trastuzumab monotherapy in breast cancer patients (48). However, both drugs significantly alleviated the cardiotoxicity of anthracycline and trastuzumab sequential therapy. Although, ARBs, ACEIs, and β-blockers are necessary for treatment of HF, long-term studies are essential to validate whether ARBs have cardioprotective effects against the chronic or late-onset types of cardiotoxicities induced by cancer treatments.

### Newly Discovered GPCR Agonist Against Anticancer-Mediated Cardiotoxicity

**Figure 1:**
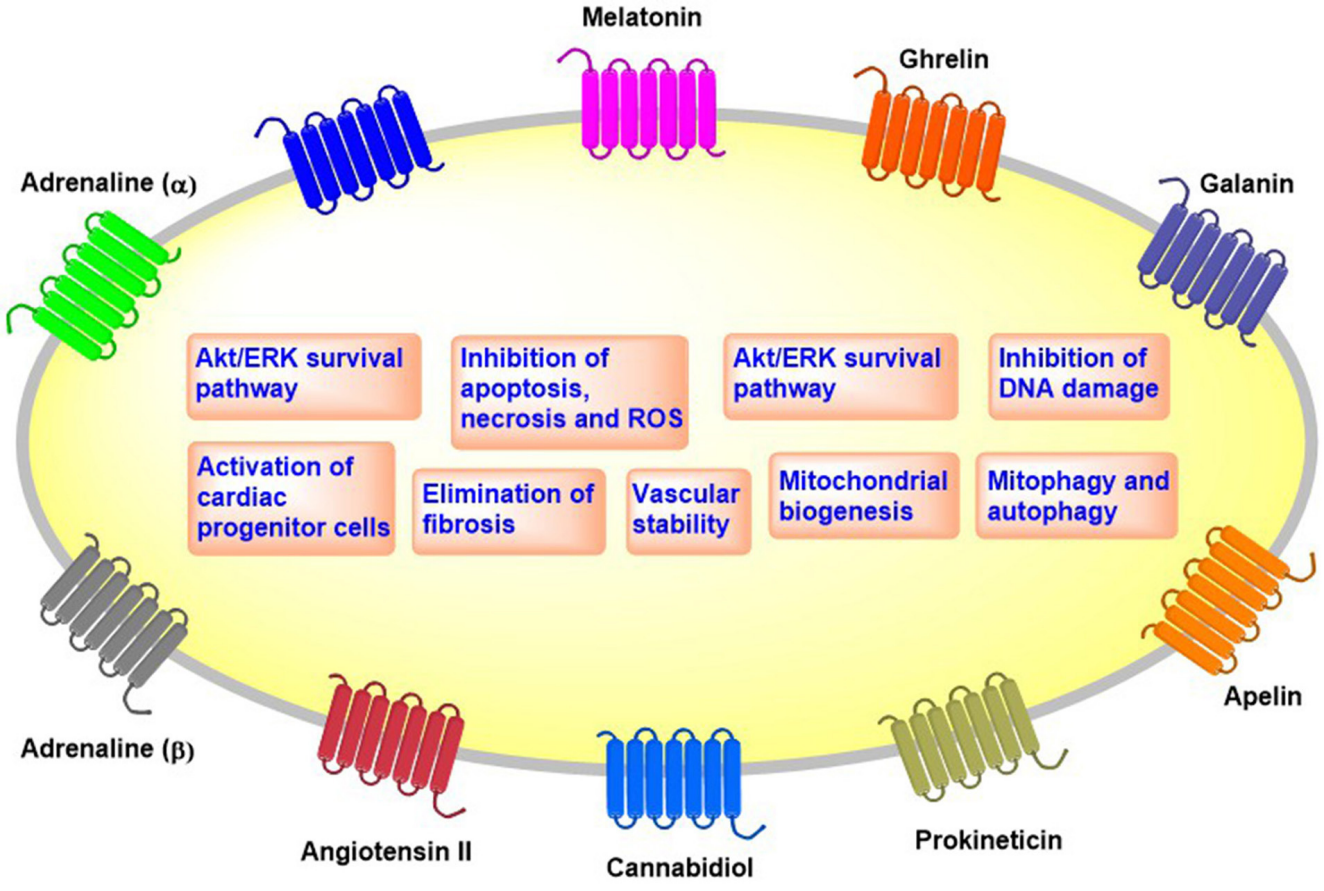
Overview of the cellular effects of cardioprotective GPCRs.

We discus here newly identified GPCR agonists that exhibit cardioprotective effects against anti-cancer drugs in *in vitro* and *in vivo* preclinical models (Figure 1 and Table 2).

**Table 2:** Newly discovered cardioprotective agents targeting GPCRs.

**Newly discovered cardioprotective agents targeting GPCRs**	**Mechanism of cardioprotection against anticancer-mediated cardiotoxicity**	**Name of molecules**	**Tumor effect**	**Study limitations**
**Alpha adrenergic receptor** (α 1AR) Via Gα_q_/G_11_ ⇑PLC/Ca^+2^	⇓ROS,⇑ mitochondrial function, ⇑ATP content, ⇑ERK 1/2 phosphorylation (84)	Dabuzalgron α 1AR agonist	No effect on anticancer efficacity in animal models (84)	While dabuzalgron a well-tolerated oral α1A-AR agonist, there has been no clinical trial on its cardioprotective role yet
**Adenosine** (A_1_R and A_3_R) Via Gα_i/o_ ⇓ cAMP /PKA /CREB. Via Gαq ⇑PKC ⇓cardiac K^+^ channels and voltage sensitive Ca^2+^ channels	⇓oxidant/⇑antioxidant ⇓inflammation, ⇓K_ATP_ channels,⇑neovascularization (85, 86)	Neladenoson (BAY 1067197) A_1_AR agonist Cl-IB-MECA CP-608,039 34 CP-608,039 35 A3AR agonist	Highly selective receptor subtype agents are necessary Their effects on anticancer efficacity is not known	Multiple clinical trials with two A_3_AR agonists are ongoing
**Melatonin** (MT1 and MT2) MT1 via Gαi ⇓AC/AMPK/PGC1α, ⇑PLC/PKC via Gαq. MT2 couples Gαs They dimerize with 5-HT_2c_, GPR61, GPR62, GPR50, GPR135	⇓ROS ⇓mitochondrial permeability transition pore (mPTP) ⇓ lipid peroxidation (87–93)	Circadin ^TM^ Country Life^®^ Melatonin	Melatonin increases anticancer efficacity of anthracycline in animal models (93)	Receptor oligomerization may contribute to the functional diversity of Melatonin It needs to be further exploded in human trials
**Ghrelin** (GHS-R)⇑PI3K, Akt, and NOS and p38-MAPK and ⇓AMPK activity.It dimerizes with SSTR5, DR2, MC3R, 5-HT2C	⇑Autophagy ⇓ROS and mTOR induction (94, 95)	Hexarelin and GHRP-6 agonist	The role of ghrelin administration on antitumor efficacity of anticancer drugs is not known	Receptor oligomerization may contribute to the functional diversity of ghrelin Clinical trials are needed
**Galanin** (GalR1, 2, 3) GalR1-3 couple to Gαi/Gαo, ⇑Rho	⇑ Functional and metabolic tolerance of the heart (96, 97)	GalR1-3 agonist Spexin (GalR3 agonist)	The role of galanin administration on antitumor efficacity of anticancer drugs is not known	It needs to be further exploded in human trials
**Apelin** (APJ) ⇑AMPK and PI3K, and MAPK/ERK kinase 1/2	⇓ROS and SOD ⇓DNA damage ⇓PARP cleavage and caspases activation (98, 99)	Apelin-13 (APJ agonist)	The role of apelin administration on antitumor efficacity of anticancer drugs is not known	It needs to be further exploded in human trials
**Prokineticin** (PKR1 and PKR2) PKR1 couple to Gαq/11 activates Akt, MAPK, detoxification pathways. PKR2 couple to Gα_12/13_ and Gs.	⇓ROS, ⇑detoxification sytem, ⇓DNA damage, ⇓Cleavage of caspases Protects endothelial cells, cardiomyocytes and cardiac progenitor cells via Akt and MAPK activation (100)	IS20, PKR1 agonist	It does not alter anti-tumor efficacity of chemotherapeutics in animal models (100)	It needs to be further exploded in human trials
**Cannabidiol** (CB_1_ and CB_2_) CB1 couples to Gαi/o, CB2 couples to Gα_s_ and activates MAPK, inhibit Na^+^/Ca^2+^ exchange It activates GPR55, TRPV1, α_1_-AR, μ opioid and 5HT_1*A*_	⇓ ROS and RNS, ⇑mitochondrial function ⇓ inflammation (101, 102)	Rimonabant, AM281 (CB1 receptor antagonist), AM1241 and JWH-133 (CB2R agonist)	Cannabidiol has antitumor effects in a large variety of cancer cell lines (103)	Cannabidiol can be used glioblastoma multiforme and childhood epilepsy in humans Receptor oligomerization should be clarified

#### Alpha Adrenergic Receptor (Dabuzalgron)

Both the adrenergic receptors alpha 1 (α-AR1) and alpha 2 (α-AR2) bind catecholamines (epinephrine and norepinephrine). The α-AR1 couples to Gαq type, resulting in activation of phospholipase C, increasing Inositol trisphosphate (IP3) and diacylglycerol (DAG), and ultimately increasing the intracellular Ca^2+^ levels, leading to smooth muscle contraction and glycogenolysis (104). Cardiac α_1_-ARs activate phospholipase C and MAPK to promote ischemic preconditioning (105), cardiac hypertrophy (106)and cardiac cell survival (107). The knockout of α_1A_/α_1B_-adrenoceptor in mice develops small hearts (108) and aggravates the pressure overload–induced HF. In support of this study a large-scale clinical trial showed that doxazosin, an inhibitor of α-AR1 signaling, increases HF in hypertension patients (109). The α2-AR acts via Gαi/o to an inhibit adenylyl cyclase, decreasing the available cAMP (110). It also decreases neurotransmitter release and central vasodilation.

Dabuzalgron is a selective α1AR agonist that has been clinically examined against urinary incontinence (111). Recent study in mice showed that dabuzalgron displayed a strong cardioprotection against DOX-induced cardiotoxicity (84). It reduces ROS production and fibrosis, enhances contractile function, and preserves myocardial ATP content via regulating mitochondrial function, in DOX-treated mice. Cardioprotective signaling pathways of α1-AR is not limited to activation of MAPK1/2 pathways (84), it also activates pro-survival pathways such as A kinase anchoring protein-Lbc (AKAP-Lbc) and its anchored protein kinase D1 (PKD1) in cardiotoxicity mice models (112). Future studies should determine whether dabuzalgron can be used to treat chemotherapeutics-mediated HF in cancer patients.

#### Adenosine Receptor Agonists

Adenosine is a naturally occurring nucleoside formed by the degradation of ATP. Extracellular adenosine concentrations rise in response to hypoxia and other stress (113). However, chronic adenosine elevation can increase inflammation, cytokine release, and induces brain dopamine depletion, fibrosis and kidney damage (114). The adenosine receptors A_1_R, A_2A_R, A_2B_R, and A_3_R can sense an imbalance of demand and supply of oxygen and nutrients (115). Adenosine exerts a significant cardioprotective effect during cardiac ischemia by activation of the A_1_R and A_3_R (86, 116). However, full A_1_R agonists have promote several cardiovascular adverse effects due to its off-target activation as well as desensitization of A_1_R, leading to tachyphylaxis (117). In contrast, a selective partial agonist for A_1_AR improves cardiac function without promoting atrioventricular blocks, bradycardia, or unfavorable effect on blood pressure (118, 119).

A selective A_3_R agonist (Cl-IB-ME) mitigates bradycardia, elevated serum creatine kinase levels and cardiac histopathological changes in DOX-treated mice. Cardioprotective effect of Cl-IB-ME involves the inhibition of ROS production and inflammation induced by DOX *in vivo* (85). A_3_AR activation also prevents perioperative myocardial ischemic injury (120), protects ischemic cardiomyocytes by preconditioning (121), and induces ischemic tolerance that is dependent on K_ATP_ channels (122). This cardioprotective effects A_3_R agonists were absence in A_3_AR deficient mouse cardiomyocytes, showing an A_3_AR-mediated effect. On the opposite to A_1_AR, A_3_AR is expressed at very low levels in adult ventricular cardiomyocytes. The efficacy of two A_3_AR agonists is currently examined in multiple clinical trials (123).

#### Melatonin Receptor Agonists

Melatonin is a pineal gland hormone synthesized from the amino acid tryptophan and is secreted into both the bloodstream and cerebrospinal fluid. It regulates circadian, seasonal, and transgenerational time cycles. Melatonin acts through 2 GPCRs, MT1, and MT2 that are linked to Gα_i_/Gα_o_ or Gα_q_/Gα_11_ to induce anti-adrenergic effects (124). These melatonin receptors are ubiquitously present in central and peripheral organs, including the cardiovascular system. Melatonin regulates blood pressure and heart rate either normalizing the circadian rhythm of blood pressure and ameliorating nocturnal hypertension, or directly acting on heart and blood vessels (125). They also regulate the renin-angiotensin system (126) and mitochondrial function (127).

Melatonin inhibits necrosis and apoptosis, and improves DOX-mediated cardiac dysfunction without compromising the antitumor effect of DOX in mice (87) and rats (88). The mechanism involved in cardioprotective effect against DOX-cardiotoxicity has been attributed to its antioxidant effect (89) and suppression of lipid peroxidation (90). Recent studies showed that melatonin activates AMPK, PGC1α (91), and sirtuins (92) to attenuate acute DOX-cardiotoxicity via alleviating mitochondrial oxidative damage and apoptosis. Indeed, high doses of melatonin are essential to reach adequate subcellular concentrations to exert these cardioprotective effects (128).

Ramelteon, is a dual MT1 and MT2 melatonin receptor agonist used for insomnia that displays a strong cardioprotective effect in the models of ischemic HF induced by the coronary artery ligation (129), chronic intermittent hypoxia-induced HF (130), and isoproterenol-induced myocardial infarction (131, 132). Unfortunately, the effect of ramelteon in anticancer-mediated cardiotoxicity has not been studied yet. Melatonin can also enhance antitumor effects of anthracycline in animal model (93). Thus, the combined treatment of anthracyclines and melatonin needs to be further explored in cancer patients.

#### Ghrelin Receptor Agonists

Ghrelin is a growth hormone-releasing and orexigenic peptide that acts through growth hormone secretagogue receptor (GHS-R) in the brain. However, expression of GHS-R in cardiovascular system is controversial. Ghrelin regulates energy balance, body weight maintenance, and metabolism (133). Roles of ghrelin in protecting heart function and reducing mortality after myocardial infarction are partly due to its role on the cardiac vagal afferent nerve terminals (inhibition of cardiac sympathetic and activation of cardiac parasympathetic nerve activity) (134). Ghrelin significantly decreased blood pressure and heart rate in healthy human (135) and prevents the arrhythmia in the mice model of myocardial infarction (136).

Ghrelin significantly improves LV functions and attenuates fibrosis (137) and development of cachexia (138) in rat HF model. Ghrelin inhibits the DOX -induced cardiotoxicity in mice hearts and cardiomyocytes by blocking AMPK activity and activating the p38-MAPK pathway, which suppresses excessive autophagy (94). A ghrelin-containing salmon extract given per os was found to alleviate the cardiotoxicity of DOX in mice, mimicking cardioprotective effect of synthetic ghrelin (95). Cardioprotective effect of ghrelin can also be due to its angiogenic properties in ischemic tissue (139–141). Ghrelin via GHS-R ameliorates impaired angiogenesis by increasing VEGF levels in the ischemic hearts of diabetic rats (140) and in a rat myocardial infarction model (142). Despite the potent synthetic agonist of GHS-R, RM-131 plays an anticatabolic effect in chronic HF models of rat (143), its role in anti-cancer drug mediated cardiotoxicity has not been studied yet.

#### Galanin Receptor Agonists

Galanin is a neuropeptide present in the nervous system and some organs (144) that uses 3 kinds of GPCRs called GalR1, GalR2 and GalR3 that are all expressed in the cardiovascular system (145). The elevated sympathetic activity during cardiac failure stimulates the release of galanin. This neuropeptide is a one of the sympathetic co-transmitters together with ATP and neuropeptide Y (NPY), in addition to norepinephrine. Galanin released by sympathetic nerves may diminish vagal neurotransmission (146). Indeed, galanin via GalR1 inhibits vagal bradycardia (147). In accord with this study, GalR1 inhibitor, M40 improves cardiac function and attenuate remodeling after myocardial infarction in rats (148). In contrast, an peptide agonist of galanin receptors and the full-length galanin reduce infarct size and the cardiac damage markers in ischemia and reperfusion rat model (96). Indeed, the natural N fragments of Galanin that have more affinity to GalR2 than GalR1 and GalR3 (145) limit acute myocardial infarction in rats *in vivo* (149). Moreover, natural galanin and GalR2 agonist have shown to increase cell viability by suppressing caspase-3 and 9 activity against hypoxic insults in other cells (97).

The GalR1-3 agonist [RAla14, His15]-galanin (2-15) exhibits cardioprotective properties against DOX-mediated cardiac injury in rats. Coadministration of this agonist with DOX has prevented the increase in plasma CK-MB activity and improved the parameters of cardiac function and caused weight gain. The obtained results demonstrate the ability of a novel agonist of galanin receptors GalR1-3 to attenuate DOX-induced cardiotoxicity (150). To conclude, galanin peptides via GalR1-3 alleviate the cardiac dysfunctions induced by DOX. The role of GalR1-3 agonist on anti-tumor effect of DOX in cancer mice model needs to be studied.

#### Apelin Receptor Agonists

Apelin is an endogenous peptide that acts trough the APJ receptor that is 54% identical with AngII receptor. However, angiotensin II does not bind to APJ (151). Mature apelin, apelin-36, and its shorter forms (apelin-17, -12, and -13) result from the cleavage of pre-pro-apelin. Apelin itself can also be cleaved *in vitro* by the angiotensin-converting enzyme 2 (ACE2) (152). Apelin has a positive inotropic effect *in vitro* (153) and is involved in lowering arterial blood pressure (154), inducing arterial vasodilation (155), and improving cardiac output (156). It protects the heart against ischemia/reperfusion-mediated injury and promotes angiogenesis (157).

Moreover, in APJ knockout mice exhibited more severe heart injury, including impaired contractility functions and survival rate after DOX treatments as compare to wild type mice receiving DOX (98). On the other hand, apelin protects H9c2 cardiomyocytes overexpressing APJ against DOX-mediated cell death. These findings all together have suggested that the suppression of APJ expression can worsen DOX-induced cardiotoxicity. Impairment of the endogenous apelin-APJ system may partially depress the protective signaling in DOX-treated hearts (98). Apelin-13 pretreatment attenuates cisplatin-induced cardiotoxicity by inhibiting apoptosis in cardiomyocytes via activation of MAPKs and PI3K/Akt signaling *in vitro* and *in vivo* in mice heart (99). The mechanism of cardioprotection *in vivo* involves an attenuation of the ROS and superoxide anion accumulation, inhibition of DNA damage, and suppression of PARP and caspases as well as an improvement in angiogenesis.

Importantly, high levels of apelin and APJ have been found in several cancer types that may be connected with obesity. For example, increase levels of Apelin-12 in colon cancer patients with obesity (158), or elevated levels of apelin-36 in endometrial and breast cancer patients with obesity (159–161) have been found. The role of AJP agonist on anti-tumor effect of anti-cancer agents in cancer mice model needs to be studied. Thus, promoting APJ signaling in heart may represent an interesting strategy to alleviate the cardiotoxicity of anticancer treatments.

#### Prokineticin Receptor Agonists

Prokineticins are peptides found in milk and macrophages (162). These peptides are called prokineticin because of their first identified biological activity was a prokinetic effect on smooth muscle cells of the gastrointestinal tract (163). Prokineticins exist as two isoforms, PROK1 and PROK2 that are expressed in all mammalian tissues (164). They are angiogenic factors (165) and induce mitogenic and survival pathway in lymphocytes and hematopoietic stem cells (166), neuronal cells (167, 168), cardiomyocytes (169), and endothelial cells (170). PROK1 and PROK2 exert their biological activity on prokineticin receptors 1 and 2 (PKR1 and PKR2) (171).

We have showed that PROK2/PKR1 can induce angiogenesis, while PROK2/PKR2 signaling promotes endothelial cell fenestration and disorganization (170). In cardiomyocytes PKR1 signaling activates Gα_11_/Akt pathway to reduce cardiomyocyte death (169), while PKR2 signaling induces hypertrophic cardiomyopathy (172). Indeed, PKR1 gene therapy promotes resistance to ischemia, protects heart against myocardial infarction, and ameliorates heart structure and function (169). Overexpression of PKR1 in transgenic mice hearts promotes neovascularization, suggesting a novel myocardial-epicardial interaction that is involved in differentiation of epicardial progenitor cells (EPDCs) in to vasculogenic cells type by a paracrine PROK2/PKR1 signaling (173).

PKR1 signaling controls epithelial mesenchymal transformation (EMT) during heart (174) and kidney development (175). PKR1 controls fate of tcf21^+^ fibroblast (176) and Wt1^+^ epicardial cells (174). PKR1 epigenetically controls stemness and differentiation of these cells, unraveling a new neovasculogenic pathway vs. adipogenesis (177). PKR1 inhibits adipogenesis and reduce adipocyte accumulation under high fat diet regime of mice (178, 179). PKR1 controls trans-endothelial insulin uptake, preadipocyte proliferation and adipogenesis (180). Lack of PKR1 in mice induces developmental defect in heart and kidney and in adult stage insulin resistance and obesity (181, 182).

In 2015, Gasser et al. discovered the first PKR1 agonists called IS20 (183). This agonist prevents the formation of cardiac lesions and ameliorates the cardiac function and survival after myocardial infarction in mice. IS20 inhibits DOX-mediated cardiotoxicity in cultured cardiac cells including cardiomyocytes, endothelial and progenitor cell as well as in mice models of acute and chronic cardiotoxicity. Importantly, these small molecules did not alter cytotoxic effect of DOX in cancer cells and *in vivo* cancer cell line- derived xenograft mice model (100). This study also described how classic chemotherapeutics, anthracyclines affect cardiac cells in dose-and time-dependent manner and how they impair NFR2 defense mechanism. These results indicate that PKR1 is a target for development of cardioprotective drugs.

#### Cannabidiol

Cannabidiol is the most abundant non-psychoactive, derived cannabinoid (184). In the low nanomolar range, cannabidiol act as an antagonist of cannabinoid 1 receptor (CB_1_R) and cannabinoid 2 receptor (CB_2_R), while it has agonist/inverse agonist actions at micromolar concentrations (185, 186). Cannabidiol activate TRPV1 channel and several GPCRs, including the orphan receptor GPR55, the putative Abn-CBD receptor, α_1_-adrenoreceptors, 5HT_1A_ receptors and μ opioid receptors (187). Several studies showed cardioprotective effects of cannabidiol in animal models of myocardial ischemic reperfusion injury (188), and myocardial infarction (189). It also ameliorates cardiac functions in diabetic cardiomyopathy (186).

Cannabidiol protects hearts against DOX-induced cardiac injury, in rats (101) and in mice (102). It improves cardiac dysfunction by (i) attenuating ROS /RNS accumulation, (ii) preserving mitochondrial function and biogenesis, (iii) promoting cell survival, and (v) decreasing myocardial inflammation. The involvement of CB_1_ and CB_2_ signaling were not clarified in these studies. Recent data has shown that CB_1_R and CB_2_R receptors have opposite effects. Indeed CB_1_R antagonists and CB2R agonists both protect the heart against clozapine-toxicity (190). Thus, CB_1_R antagonist reduces DOX-induced cardiotoxicity and decreased cortical cerebral infarction (191). By contrast, two CB_2_R agonists JWH-133, AM 1241 alleviate quetiapine cardiotoxicity (192). Moreover, cannabidiol by itself display cytotoxicity in many cancer cell lines, and anti-tumor effects in cancer mice models (103), suggesting that cannabidiol may have a synergistic effect with antineoplastic drugs in the use of cardioprotective agents. In fact, the cannabinoid HU-331 has been shown to be more potent and less cardiotoxic than DOX (193). Indeed, Insys Therapeutics has obtained FDA orphan drug designation for Cannabidiol for the treatment of multiform glioblastoma and childhood epilepsy.

## Conclusion

Cardiotoxicity induced by anti-cancer therapy may occur when the anticancer agent targets a common signaling pathway that are essential to maintain the functions of both cardiac and cancer cells. It can also involve off-target effects due to non-selective actions of anti-cancer agents. The choice of the cardioprotective therapeutic approach relies on the delicate balance between the efficiency of anti-neoplastic drugs and the management of cardiovascular complication.

**Figure 2:**
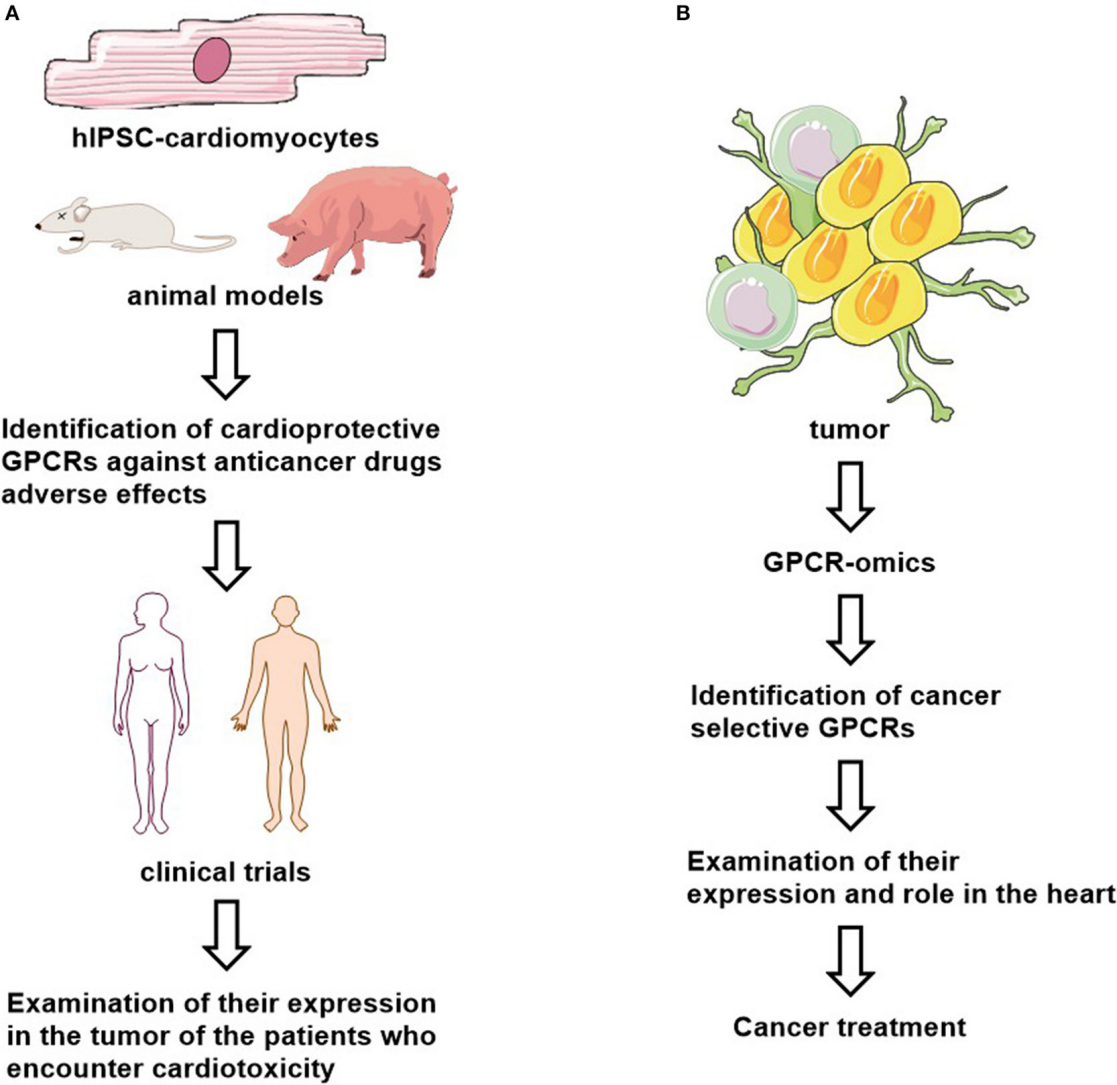
Proposed cardioprotective **(A)** and anti-cancer **(B)** drug studies.

Cardioprotective utility of GPCR ligands will require validation of preferentially expression of these GPCRs in both cancer and cardiac cells, and identification of their signaling (e.g., G-protein- or arrestin-mediated pathways) and functional roles (Figure 2A). Whether these cardioprotective ligands interfere with the anti-tumor effect of the chemotherapeutics should be studied as well. The human inducible pluripotent stem cell derived cardiomyocytes (hiPSC-CMs), iPSC-CM-derived 3D cultures and organoids provide human-based model systems to explore the molecular mechanisms of cardiotoxicity and cardioprotection (194). They may also serve as a platform for personalized medicine. Thus, GPCR ligand efficacy can be optimized and their side-effects can be examined in hiPSC-CMs and organoids.

In addition, most of the data regarding the efficacy of cardioprotective GPCR-ligands against cancer therapy mediated-cardiotoxicity have been obtained from small animal models of cardiotoxicity and cancer cell-derived xenograft mice models. Therefore, further studies in bigger animals are necessary to examine their efficacy and adverse effects before these findings can be translated to a human study.

Interestingly, certain cancer cell types may retain a GPCR expression pattern via serving novel biomarkers and/or as valuable therapeutic targets. For example, GPR161 is functionally expressed in breast cancer (195) and GPRC5A in pancreatic cancer (196) and GPR68 in the tumor microenvironment (197). However, both CD97 and GPR56 are highly express in multiple cancer types and in normal tissues (198). Moreover, many mutated GPCRs such as GPR110, GPR112, GPR125, GPR126, GPR98, and GPR110 have been found in certain cancers (199). These findings suggest that different types of cancers may be characterized by a specific onco-GPCR-ome (67). It could be interesting to examine if there is a “GPCR signature” in heart as well. In precision medicine, selectively targeting GPCRs in specific cancers can lead to a novel class of anti-cancer drugs with less adverse cardiac effects, after defining their expression and their role in heart (Figure 2B).

## Author Contributions

AA, LD, and CN participated in writing and drawings of the manuscript.

### Conflict of Interest

The authors declare that the research was conducted in the absence of any commercial or financial relationships that could be construed as a potential conflict of interest.

## Abbreviations

GPCR: G protein-coupled receptor
DOX: Doxorubicin
HF: Heart failure
ROS: Reactive oxygen species
RNS: Reactive nitrogen species
Top Iiβ: Topoisomerase I*i*β
LVEF: LV ejection fraction
HER2: Hergulin2
CTLA-4: T-lymphocyte associated antigen-4
PD-1: Programmed cell death protein-1
LDL: low-density lipoprotein
HMG-CoA: 3-hydroxy-3-methyl-glutaryl-coenzyme A reductase
PI3K: Phosphoinositide 3-kinases
MAPK: Mitogen-activated protein kinases: β-ARs: β-adrenergic receptors: ? -ARs???-adrenergic receptors
CaMKII: Calmodulin-dependent protein kinase II
Ang-II: Angiotensin II
AT-1R and AT-2R: Angiotensin receptors
RAS: Renin-angiotensin system
ARB: Angiotensin-II receptor blockers
IP3: Inositol trisphosphate
DAG: Diacylglycerol
PKD1: Anchored protein kinase D1
ATP: Adenosine-triphosphate
A_1_R, A_2A_R, A_2B_R and A_3_R: Adenosine receptors
MT1 and MT2: Melatonin receptors
mPTP: Mitochondrial permeability transition pore
GHS-R, Ghrelin receptor: growth hormone secretagogue receptor
VEGF: vascular endothelial growth factor
GalR1, GalR2 and GalR3: Galanin receptors
APJ: Apelin receptor
ACE2: Angiotensin-converting enzyme 2
PARP: Poly(ADP-ribose) polymerase
PROK1 and PROK2: Prokineticins 1 and 2
PKR1 and PKR2: Prokineticin receptors
hiPSC-CMs: Inducible pluripotent stem cell derived cardiomyocytes.
